# Correlation of temperament characteristics and early functional exercise compliance in school-age children with limb fractures: Implication for clinical nursing care

**DOI:** 10.1097/MD.0000000000032305

**Published:** 2022-12-30

**Authors:** Ping Wu, Shangju Chen, Yi Gu, Yuping Tang

**Affiliations:** a Department of Orthopedics, Children’s Hospital of Nanjing Medical University, Nanjing, Jiangsu Province, China.

**Keywords:** care, children, compliance, fractures, functional exercise, nursing, temperament

## Abstract

Early functional exercise plays a very important role in the rehabilitation and nursing care of children with fractures. We aimed to evaluate the role of temperament characteristics in the early functional exercise compliance in school-age children with limb fractures, to provide evidence to clinical nursing care. School-age children with limb fractures admitted to our hospital from January 1, 2021 to June 30, 2022 were selected. We collected the general information, evaluated the temperament characteristics and their functional exercise compliance in school-age children. Correlation and regression analyses were conducted to assess the correlation of temperament characteristics and early functional exercise compliance. A total of 126 school-age children with limb fractures were finally included. There were 68, 36, and 22 children that were rated as easy-going, troublesome and initiate slow temperament, respectively. The children with easy-going temperament had the best early functional exercise compliance, followed by children with initiate slow temperament, and compliance of children with troublesome temperament was the worst. The reaction intensity factor in the temperament characteristics of school-age children with fracture was negatively correlated with early functional exercise compliance (*P* = .007). Reaction intensity was a risk factor for early functional exercise compliance in school-age children with fractures (*P* = .004). Health care providers must consider the influence of temperament characteristics on compliance in school-age children with fractures, and take targeted nursing measures according to different temperament characteristics of children to improve early functional exercise compliance in school-age children with fractures, so as to improve the functional recovery and prognosis of children.

## 1. Introduction

Accidental injury is one of the health problems that leads to the decline of children’s quality of life and endangers children’s lives, which is also a global public health problem. Children’s fractures account for 33.5% of all accidental injuries.^[1,2]^ Children’s psychological and behavioral development is more mature than that in early childhood, but their experience in life, self-protection ability, concentration and self-control are still not strong.^[3]^ When children enter school life at this stage, their parents’ awareness of protection is more relaxed than before, and school-age children’s self-safety awareness is not strong, making the school-age children prone to upper or lower limb fractures.^[4,5]^ In addition to correct treatment after fracture, the functional exercise of the affected limb after treatment is very important. Previous studies^[6,7]^ have shown that early functional exercise of the affected limb after a fracture can promote blood circulation in the affected limb, reduce swelling of the affected limb, and prevent the occurrence of compartment syndrome. However, in the early stage of the fracture, the affected limbs of the children will experience discomfort such as pain, swelling, and mobility dysfunction. The hospital is an unsafe and unfamiliar environment, increasing the possibility that the children will feel nervous, fearful and even crying.^[8]^ There are still often cases where the child does not cooperate with the treatment and nursing plans and functional exercises. Therefore, how to increase the compliance to functional exercise of children after limb fracture is a current focus for pediatric fractures treatment and nursing care.

Temperament characteristics belong to the psychological characteristics of individuals, and are a manifestation of behavioral externalization, that is, the way individuals change their emotions in the process of responding to the external environment.^[9]^ Jaruseviciute et al have reported that temperament is the basis of personality development, which can directly affect the individual’s psychological activities and behaviors, and interact with their physical diseases.^[10]^ Ju et al^[11,12]^ have reported that the mutual influence of children’s temperament and physical diseases can be divided into 2 categories. On the one hand, it affects the problems and conditions that occur in the process of children’s growth, and on the other hand, it affects children’s processing and results of certain events. With the in-depth study of temperament characteristics in medicine, it is found that temperament can affect various aspects of children’s life, study, education and disease. Currently the relationship between temperament characteristics and early functional exercise compliance in school-age children with fractures remains unclear. Therefore, this study aimed to investigate the correlation between temperament characteristics and early functional exercise compliance in school-age children with limb fractures, and to provide reference and evidence support for improving early functional exercise compliance in school-age children with fractures.

## 2. Methods

This study was a cross-sectional survey design. The study protocol had been approved by the ethical committee of Children’s hospital of Nanjing medical university (approval number: 202205089-1), and written informed consents had been obtained from the legal guardians of children.

### 2.1. Study population

School-age children with limb fractures admitted to our hospital from January 1, 2021 to June 30, 2022 were selected as the research population. The inclusion criteria were: School-aged children aged 7 to 12 years; The children have basic comprehension ability and can understand the content of the questionnaire; The children and guardians agree to participate in the study and signed the informed consents. Exclusion criteria were: Those children with dementia or brain lesions without normal cognition and comprehension ability; Children hospitalized for less than 3 days; Children who did not agree to participate in this study.

### 2.2. Exercise

The functional exercise started as early as possible. We started the upper functional exercise of fist clenching and fist releasing on the affected limb at the early stage to make the whole upper limb muscles relax and contract (fist clenching and fist releasing are once), 4 times per minute, 3 times a day, 5 to 10 minutes each time; When moving the shoulder joint, bend 90°, the children used one hand to drag the forearm of the affected limb for circular motion of the shoulder joint. The nurse instructed the family members to assist the patients in functional exercise on time. Three to 5 weeks after injury. In addition to practicing the muscle contraction and relaxation of the affected limb, we guided the children to move the joints near the fracture and gradually increase the intensity of exercise. The upper limb fracture could be used for active joint extension and flexion activities, such as wrist joint, elbow joint, extension and flexion, abduction and adduction of the whole upper limb. The lower limb exercises were: the children carried out bed exercise activities such as hip joint adduction and external rotation flexion and extension, knee joint flexion and extension training, paid attention to maintaining the operating limb in the external booth, control the hip joint internal flexion less than 90°, and increased it according to the situation. When going upstairs, the healthy limb should go up first, and when going downstairs, the affected limb should go down first. At 2 months after the operation, the main exercise contents were straight leg lifting, single leg balance and hip joint extension, 10 times a day for 1 minute each time, until the lower limb of the affected side could complete the task of standing alone, and then walking with crutches as an assistant.

### 2.3. Survey

The unified and specially trained nurse communicated with the child and the family members or main caregivers who know the child best, because the family members and main caregivers who know the child best have a long contact time with the child, and observe the behavior of the child for a long time and carefully. The 2 researchers explained the purpose of the questionnaire to the children, their families and the main caregivers. After obtaining the consent of the children and their families, they explained the questions and filling requirements of the questionnaire in detail, so that the children and their families could fully understand the questionnaire. After understanding, the family members and guardians filled in the temperament scale. The early functional exercise compliance evaluation form for children with school-age fractures was conducted by specially trained nurses using a combination of direct observation and interviews to evaluate the early functional exercise compliance of school-age children with fractures.

We reviewed literature and consulted relevant experts, and used self-designed questionnaires to collect general information of children, including age, gender, body mass index, place of residence, type of fracture, site of fracture, causes of fracture.

#### 2.3.1. School-aged children’s temperament scale.

This Chinese version of scale^[13,14]^ includes 9 temperament factors including activity level, rhythmic, reaction intensity, adaptability, mood, persistence, avoidance, reaction cutoff, attention diversion, with a total of 100 items, using a 6-point scoring system from 1 to 6 points. The temperament types are divided into 3 types: easy-going temperament, troublesome temperament and initiate slow temperament. This scale has been widely used for school-aged children’s temperament with good reliability and validity.^[15]^

#### 2.3.2. Exercise compliance scale.

This Chinese version of scale^[16]^ includes when to exercise early, whether to exercise according to the number of times the nurses instructed, whether to exercise according to the nurse’s guidance method, whether to exercise according to the time instructed by the nurses, and whether to exercise the affected limb during the early functional exercise. Whether the pain will stop exercising has 5 dimensions, using a 5-point scoring system from 1 to 5 points. in which a score of ≤ 13 points indicating noncompliance, 14 to 20 points indicating partial compliance, and ≥ 21 points indicating full compliance. The internal consistency reliability coefficient of this scale is 0.8812, and the test-retest reliability coefficient is 0.8165, indicating this scale has good reliability and validity.^[17]^

### 2.4. Statistical methods

In this study, SPSS 23.0 statistical software was used for data analysis. We reviewed the collected data, and used Excel software to build a database. We used chi-square test, analysis of variance, correlation analysis, logistic regressions and other statistical methods to analyze the correlations of temperament characteristics and exercise compliance in school-age children with limb fractures. The test level of this study was *α* = 0.05, and *P* < .05 was considered as a statistically significant difference between groups.

## 3. Results

### 3.1. Characteristics of included children

A total of total of 135 children were initially included, 9 children were transferred to other areas or hospital without complete data. Therefore, 126 school-age children with limb fractures were finally included, involving 55 female children and 71male children. The proportion of children with fractures around the age of 10 years old was relatively large, and most of them are upper limb fractures. The characteristics of included children were presented in Table 1.

**Table 1:** Characteristics of included children (n = 126).

Characteristics	Variables
Age (y)	10.22 ± 3.56
Female/male	55/71
BMI (kg/m^2^)	21.34 ± 2.44
Only child of family	
Yes	49(38.89%)
No	77(61.11%)
Place of residence	
Rural area	44(34.92%)
City	82(65.08%)
Guardians	
Parents	82(65.08%)
Grandparents	34(26.98%)
Others	10(7.94%)
Type of fracture	
Closed	88(69.84%)
Open	38(30.16%)
Site of fracture	
Upper limb	75(59.52%)
Lower limb	40(31.75%)
Both	11(8.73%)
Causes of fracture	
Traffic accident	66(52.38%)
Fall	56(44.44%)
Others	4(3.17%)
Type of temperament	
Easy-going temperament	68(53.97%)
Troublesome temperament	36(28.57%)
Initiate slow temperament	22(17.46%)

BMI = body mass index.

### 3.2. Temperament characteristics

Of the included 126 children, there were 68 children that were rated as easy-going temperament 36 children were rated as troublesome temperament, 22 children were rated as initiate slow temperament. As showed in Table 2, there were significant differences in the scores of 7 temperament factors of 3 temperament types in school-age children with fractures: reaction intensity, adaptability, mood, persistence, avoidance, reaction cutoff and attention diversion (all *P* < .05), while the scores of activity level and rhythm were not significantly different (all *P* > .05).

**Table 2:** Temperament scores in school-age children with limb fractures.

Temperament factors	Easy-going temperament	Troublesome temperament	Initiate slow temperament	F	*P*
Activity level	3.77 ± 0.46	3.89 ± 0.44	4.08 ± 0.51	3.681	.079
Rhythmic	4.70 ± 0.62	4.82 ± 0.58	4.79 ± 0.44	3.171	.103
Reaction intensity	3.63 ± 0.49	4.94 ± 0.57	3.85 ± 0.40	4.198	.021
Adaptability	3.24 ± 0.38	3.72 ± 0.51	4.43 ± 0.51	4.227	.018
Mood	2.85 ± 0.32	3.14 ± 0.35	4.04 ± 0.48	3.184	.025
Persistence	4.18 ± 0.47	4.63 ± 0.47	5.13 ± 0.54	4.827	.009
Avoidance	3.45 ± 0.42	3.95 ± 0.52	4.45 ± 0.49	3.299	.013
Reaction cutoff	3.75 ± 0.36	3.98 ± 0.51	4.72 ± 0.53	4.007	.014
Attention diversion	3.78 ± 0.44	4.06 ± 0.36	4.69 ± 0.47	3.775	.035

### 3.3. Functional exercise compliance

Of the included 126 children, the children with easy-going temperament had the best early functional exercise compliance, followed by children with initiate slow temperament, and compliance of children with troublesome temperament was the worst. Three temperament types in school-age children with fractures had significant statistical differences in the early functional exercise compliance (*P* = .013) (Table 3).

**Table 3:** Temperament characteristics and early functional exercise compliance in school-age children with limb fractures (n = 126).

Type of temperament	Early functional exercise compliance		
	Noncompliance	Partial compliance	Full compliance
Easy-going temperament (n = 68)	4(5.88%)	33(48.53%)	31(45.59%)
Troublesome temperament (n = 36)	14(38.89%)	12(33.33%)	10(27.78%)
Initiate slow temperament (n = 22)	3(13.64%)	10(45.45%)	9(40.91%)
χ^2^	6.115		
*P*	.013		

### 3.4. Correlation analysis

As showed in Table 4, the reaction intensity factor in the temperament characteristics of school-age children with fracture was negatively correlated with early functional exercise compliance (*P* = .007), while the activity level, rhythm, adaptability, mood, persistence, avoidance, reaction cutoff and attention diversion were not significantly correlated with early functional exercise compliance (all *P* > .05).

**Table 4:** Correlation analysis of temperament characteristics and early functional exercise compliance in school-age children with limb fractures.

Temperament factors	r	*P*
Activity level	0.038	.119
Rhythmic	0.074	.201
Reaction intensity	−4.118	.007
Adaptability	0.078	.103
Mood	0.101	.095
Persistence	0.023	.206
Avoidance	0.094	.112
Reaction cutoff	0.107	.094
Attention diversion	0.056	.115

### 3.5. Influencing factors of compliance

As indicated in Table 5, regression analyses showed that reaction intensity was a risk factor for early functional exercise compliance in children with school-age fractures, while other variables including activity level, rhythmic, adaptability, mood, persistence, avoidance, reaction cutoff, attention diversion and children’s characteristics including gender, age, body mass index, only child of family, place of residence, guardians, type of fracture, site of fracture and causes of fracture did not affect compliance with early functional exercise in school-age children with limb fractures (all *P* > .05).

**Table 5:** Regression analysis on the influencing factors of compliance to functional exercises in school-age children with limb fracture.

Variables	Regression coefficients	Standard error	T	*P*
Temperament factors				
Activity level	−0.166	0.714	−0.215	.108
Rhythmic	−0.184	0.722	−0.241	.097
Reaction intensity	−2.186	0.684	−3.177	.004
Adaptability	−0.161	0.635	−2.116	.125
Mood	0.014	0.509	0.018	.116
Persistence	−0.018	0.513	−0.012	.083
Avoidance	−2.014	0.556	−0.195	.689
Reaction cutoff	−2.109	0.648	−3.114	.284
Attention diversion	−0.095	0.776	−0.126	.155
Characteristics				
Gender	0.164	0.223	0.115	.096
Age	1.189	0.215	0.204	.119
BMI	−2.058	0.209	−0.415	.086
Only child of family	−0.114	0.182	0.095	.142
Place of residence	−0.486	0.127	0.146	.099
Guardians	2.177	0.112	−0.104	.285
Type of fracture	−1.285	0.083	−0.116	.091
Site of fracture	3.118	0.190	0.284	.113
Causes of fracture	2.105	0.211	0.167	.095

BMI = body mass index.

## 4. Discussions

With the continuous development of the bio-psycho-social medical care model, people have gradually realized that the occurrence of diseases depends not only on physiological factors, but also psychological and social factors play an important role. Studies^[18,19]^ have shown that the compliance of children with fractures to the treatment and nursing plan is affected by their family members, and the temperament characteristics of children are correlated with the temperament of their family members. Children’s temperament is the basis of personality development and the main factor affecting their psychological activities and behavior. It is jointly determined by biological and environmental factors, that is, genetic factors and external environmental factors constitute each other.^[20]^ Different temperament types can have different effects on the prognosis of diseases.^[21,22]^ Therefore, understanding the temperament characteristics of school-age children with fractures plays an important role in improving children’s early functional exercise compliance. With 126 children included, we have found that the children with easy-going temperament school-age fractures had the best early functional exercise compliance, followed by children with initiate slow temperament, and troublesome temperament. Besides, the reaction intensity in the temperament characteristics of school-age children with fracture was negatively correlated with early functional exercise compliance.

Previous studies^[23–25]^ have pointed out that temperament can affect children’s behavior and has a significant relationship with the incidence of behavioral disorders. In addition, some studies^[26,27]^ have shown that the dental health of easy children is significantly better than that of troublesome children, and the number of cases of dental caries is less than that of children with troublesome characteristics. Previous studies^[28–30]^ have found that children with sleep disorders are more likely to have troublesome and slow-acting characteristics. Compared with the healthy children with normal body weight, the simple obesity children have a higher proportion of the troublesome and other negative temperament.^[31,32]^ This suggests that children’s behavior is influenced by temperament types, with the highest incidence of behavioral disturbances in children with troublesome characteristics. The results of this study showed that the 3 temperament types of school-age children with fractures had significant differences in the compliance of early functional exercise of the affected limb, indicating that the temperament characteristics of school-age children with fractures affected the compliance of children with early functional exercise. Faith and Lipsanen et al^[33–35]^ have reported that negative temperament characteristics are a risk factor for behavioral problems, while the temperament characteristics of troublesome children are mainly negative and avoidant. Some psychological factors and behavioral problems of children with both upper and lower limb fracture will affect the early functional exercise compliance of school-age children.^[36]^

Children’s behavior is directly affected by their own temperament characteristics. The compliance rate of children with easy temperament is significantly higher than that of children with troublesome and slow-moving temperament, while the compliance rate of children with troublesome temperament is significantly lower than that of children with other types of temperament. The reasons may be that children with troublesome temperament mainly adopt negative and avoidant behaviors, slow to adapt to the new environment, reduce the intensity of emotional response to pain tolerance, and affect the compliance of early functional exercise.^[37,38]^ Children with initiate slow characteristics are more sensitive to new things. Although the ability to accept and adapt to new things and the environment is not strong, but it can be accepted slowly, so the compliance is higher than that of the troublesome children. Studies^[39,40]^ have shown that children with troublesome temperament characteristics are more likely to show crying, frequent injuries, and anxiety. The stronger the response intensity, the worse the early functional exercise compliance, and the weaker the response level, the better the early exercise compliance. Reaction intensity refers to the intensity of children’s emotional reactions. Children with higher reaction intensity are more likely to be impulsive and injured, while children with low reaction intensity are more likely to take reasonable advice and not easily affected by their own emotions and external environment.^[41,42]^ Therefore, targeted intervention and nursing care measures should be taken for children with severe reaction intensity, children’s guardians and relatives should be encouraged to give more love, care, companionship to children, strengthen emotional communication and cultivate their regular and healthy lifestyle, thereby promoting the healthy behaviors of children.

The formation of children’s temperament is determined by both biological and environmental factors. Biological factors are innately determined and cannot be changed, while environmental factors are closely related to acquired interventions and educations. Studies^[43,44]^ have shown that children’s temperament is genetically determined and influenced by environmental factors, it is difficult to distinguish between the 2. The nurses can communicate with the children’s family members and main caregivers to explain that the success of the operation is only the first step to the success of the disease. Early functional exercise is necessary, and with the cooperation of the children’s family members and main caregivers, according to the temperament characteristics and the influencing factors of temperament factors in school-age children with fractures, corresponding nursing measures should be taken to improve the early functional exercise compliance of school-age children with fractures. Children with troublesome characteristics live very irregularly, avoiding and withdrawing to various stimuli, and they are slow to adapt to the new environment, but their emotional reactions are very strong and often negative.^[45,46]^ For these children, medical workers should encourage the family members of the children to communicate with the children more, care about the inner changes of the children, encourage the children to express their wishes and feelings in a correct way. Besides, appropriate rewards should be given according to the children’s behaviors, so that children can actively cooperate with functional exercise and achieve the purpose of improving children’s early functional exercise compliance. Easy-going children often have strong ability to adapt to the environment and accept new things, positive emotions, and good compliance with early functional exercise.^[47–49]^ Nurses should give more appropriate demonstrations and guidance to the children’s family members and children, and explain the importance of early functional exercise. Nurses should closely observe the functional exercise of the affected limb, observe whether the plaster of the affected limb is loose, and give timely control of the frequency and intensity of functional exercises to prevent excessive exercise and ensure the effect of functional exercise. Children with initiate slow characteristic accept and adapt to new things and the environment slowly, do not respond strongly to new things, and can adapt slowly after repeated exposure.^[50–52]^ Nurses of such children should give the children appropriate stimulation, such as watching some passionate and inspirational cartoons, listening to some uplifting music, watching some beautiful pictures, etc, to increase contact with children, and to use encouraging language increases their confidence in functional exercise, and gradually improve the compliance of children with early functional exercise.

The limitations of this study are worth considering. First, this study is a single-center cross-sectional study with a small sample size, which may lack sufficient sample power to identify other temperament characteristics that may affect children’s exercise compliance. Second, we only analyzed the correlation between early functional exercise and temperament characteristics of children during hospitalization, and did not track the correlation between long-term functional exercise and temperament characteristics after children were discharged from hospital. Therefore, future studies with larger sample size and longer follow-up are needed to further investigate the correlation of temperament characteristics and children’s exercise compliance.

## 5. Conclusions

In conclusion, we have found that among the 3 temperament types of school-age children with limb fractures, the children with easy-going temperament have the best early functional exercise compliance, followed by children with initiate slow temperament, and compliance of children with troublesome temperament is the worst. The reaction intensity in the temperament characteristics is a risk factor affecting the early functional exercise compliance of school-age children with fractures. The stronger the response intensity, the worse the early functional exercise compliance of children. In clinical nursing care practice, we need to focus on the temperament characteristics of different children and the influencing factors of temperament factors, and guide the families of children to cooperate to improve the compliance of early functional exercise and achieve better functional recovery effect.

## Author contributions

YT designed research; PW, SC, YG, YT conducted research; PW, SC, YG, YT analyzed data; PW, YT wrote the first draft of manuscript; YT had primary responsibility for final content. All authors read and approved the final manuscript.

**Investigation:** Ping Wu, Shangju Chen, Yi Gu, Yuping Tang.

**Project administration:** Yuping Tang.

**Visualization:** Yuping Tang.

**Writing – original draft:** Yuping Tang.

**Writing – review & editing:** Yuping Tang.
